# Supplementary material for: Dopamine D2-receptor blockade in humans disrupts the effect of effort on learning
Source: PLoS Biol. 2026 Apr 16;24(4):e3003765. doi: 10.1371/journal.pbio.3003765 (PMC13099096; doi:10.1371/journal.pbio.3003765)
Supplement: S1 Text — Full details of additional statistical and computational analyses, including: session effects on choice behavior; drug effects on heart rate and blood pressure; drug effects on subjective feelings; assessment of participant blinding; controlling for effects of drowsiness; controlling for effects of effort variability; testing differences between experimental blocks; testing the effect of sulpiride on choice exploration; exploring the interaction between k and φ; computational models with declining learning rates; computational models fit across both drug groups. (DOCX) [file pbio.3003765.s001.docx]

**Supplementary Analyses**

**Session effects on choice behaviour**

This study was originally conducted across two sessions, with each participant completing one placebo session and one sulpiride session (order counterbalanced across participants). In our preliminary analysis of data across both sessions, we checked whether there was a difference in learning behaviour across the two sessions. Overall, the proportion of Lose-switch behaviour was significantly lower, and the proportion of Win-stay behaviour significantly higher, in the second session compared to the first (ΔLose-switch = -0.062 ± 0.014, t(41) = 4.39, p < .001; ΔWin-stay = 0.021 ± 0.008, t(41) = -2.54, p = .015). To determine whether this session effect might confound the effects of sulpiride, we ran separate ANOVAs testing differences in Win-stay and Lose-switch behaviour by Drug (placebo or sulpiride, within-subjects), Order (placebo first or sulpiride first, between-subjects), and their interaction (refer to S1 Fig, file: S1_Fig.tif).

In both analyses, we found significant Drug x Order interactions (Lose-switch: F(1,40) = 18.5, p < .001; Win-stay: F(1,40) = 5.96, p = .019). Post hoc tests confirmed that, relative to placebo, sulpiride increased Lose-switch behaviour when taken in the first session (Order: SP → PL; ∆Lose-switch = 0.064 ± 0.02, t(40) = 3.33, p = .004), but decreased Lose-switch behaviour when taken in the second session (Order: PL → SP; ∆Lose-switch = -0.059 ± 0.021, t(40) = -2.78, p = .016). Post hoc tests of Win-stay behaviour showed that sulpiride decreased Win-stay only when the active drug was taken in the first session (Order: SP → PL; ∆Win-stay = 0.037 ± 0.01, t(40) = 3.55, p = .002) but not the second session (Order: PL → SP; p > .99).

Together, these interaction effects indicate that the effect of sulpiride was confounded by the order in which it was administered. When sulpiride was taken first, it appeared to increase lose-switch behaviour and reduce win-stay behaviour relative to placebo. However, when sulpiride was taken second, it instead appeared to reduce lose-switch behaviour while not significantly altering win-stay. These session effects on sulpiride are broadly consistent with previous work showing that the role of dopamine on reward learning can vary across different task contexts (Shiner, Seymour et al. 2012). Interpreting these longer-term order effects is further complicated by the role of dopamine in consolidating previous motor skills (Sommer, Costa et al. 2014). Given that our primary aim was to isolate the effect of effort on trial-by-trial reinforcement learning, we analysed our data with a between-subjects design, using data from the first session only (as per van Nuland, Helmich et al. 2020).

**Drug effects on heart rate and blood pressure**

Heart rate and blood pressure were measured at the start of the testing session and after every hour. We ran three mixed-effects ANOVAs to examine differences in heart rate (HR), systolic blood pressure (sBP), and diastolic blood pressure (dBP), respectively. Each ANOVA included Drug (placebo, sulpiride) as a between-subjects factor, Time (0-4 hrs) as a within-subjects factor, and their interaction (refer to S2 Fig, file: S2_Fig.tif).

There was a significant main effect of Time on HR (F(4,160) = 29.05, p < .001), such that HR decreased over the first and second hours post-ingestion across both groups (0-1 and 1-2 hrs, both p ≤ .009; 2-3 and 3-4 hrs, both p > .99). HR did not differ significantly between Drug groups (main and interaction effects both ≥ 0.53).

We also observed a main effect of Time on sBP (F(3.3,131.93) = 4.87, p = .002), such that sBP decreased in the first hour post-ingestion (0-1 hrs, p = .003) and increased in the fourth hour post-ingestion (3-4 hrs, p = .044), with no other significant changes between time points (1-2 and 2-3 hrs, both p ≥ .74). There were no significant differences in sBP between Drug groups (main and interaction effects both p ≥ 0.28). Neither Time nor Drug significantly affected dBP (all p ≥ .24).

In sum, we found no effects of sulpiride on HR, sBP or dBP compared to placebo. Small variations in HR and sBP were detected over the course of the testing session, but these changes were seen across both drug groups and remained in the normal range.

**Drug effects on subjective feelings**

At the start of the testing session and after every hour, participants used (digitised) Bond and Lader visual analogue scales (BL-VAS) to report their current subjective state. Scales measured the degree to which participants felt ‘alert/drowsy’, ‘calm/excited’, ‘strong/feeble’, ‘muzzy/clear-headed’, ‘well-coordinated/clumsy’, ‘lethargic/energetic’, ‘contented/discontented’, ‘troubled/tranquil’, ‘mentally slow/quick-witted’, ‘tense/relaxed’, ‘attentive/dreamy’, ‘incompetent/proficient’, ‘happy/sad’, ‘antagonistic/amicable’, ‘interested/bored’, and ‘withdrawn/gregarious’ (Bond and Lader 1974). In keeping with previous studies, these individual scales were used to calculate weighted averages measuring three distinct factors of ‘alertness’, ‘contentedness’, and ‘calmness’, respectively (Bond and Lader 1974, Chamberlain, Müller et al. 2006, Eisenegger, Naef et al. 2014). In addition, four scales were selected for individual analysis to aid in the detection of any possible motor changes caused by sulpiride. These were scales measuring the degree to which participants felt ‘alert/drowsy’, ‘strong/feeble’, ‘well-coordinated/clumsy’, and ‘lethargic/energetic’.

Separate mixed-effects ANOVAs were run to examine differences in these measures. Each ANOVA included Drug (placebo, sulpiride) as a between-subjects factor, Time (0-4 Hrs) as a within-subjects factor, and their interaction (refer to S3 Fig, file: S3_Fig.tif). There were no significant effects of Drug on any of the factor scores (main effect and interactions, all p ≥ .1). The ANOVA on the ‘alert/drowsy’ scale showed a significant Drug x Time interaction effect (F(2.53,101.3) = 3.52, p = .024). This was driven by small differences in the trajectory of this measure (i.e., an early peak in the placebo group and a later peak in the sulpiride group), but there were no significant differences when comparing drug groups at each individual time point (post hoc t-tests at hrs 0-4, all p ≥ .55). There were no other significant effects of Drug on the individual scales (main effect and interactions, all p ≥ .15).

**Assessment of participant blinding**

As reported in the main text, we assessed blinding efficacy with the Bang Blinding Index (S1 Table). In addition, we confirmed that the significant effect of sulpiride vs. placebo on task performance (i.e., accuracy and win-stay behaviour) was not related to participants’ awareness of their assigned drug group. We ran a two-way ANOVA testing whether Accuracy differed according to: (1) the drug that participants Guessed they had ingested (placebo, sulpiride, unsure); (2) their True Drug group (placebo, sulpiride); and (3) the Guessed Drug × True Drug interaction. In addition, we conducted the analogous ANOVA on Win-stay behaviour instead of Accuracy.

In both ANOVAs, neither the main effect of Guessed Drug, nor its interaction with True Drug, were significant (p ≥ .19). This confirms that learning performance was not affected by participants’ belief of which drug they had consumed.

| **Assignment** | **Response** |  |  |  |
| --- | --- | --- | --- | --- |
|  | Sulpiride | Placebo | Unsure | Total |
| Sulpiride | 10 | 4 | 9 | 23 |
| Placebo | 5 | 5 | 9 | 19 |
| Total | 15 | 9 | 18 | 42 |

***S1 Table. Participant blinding data.* ‘**Assignment’ refers to the true allocation of participants to the sulpiride and placebo groups. ‘Response’ refers to the participants’ belief about their allocated group (placebo, sulpiride, or unsure).

**Controlling for effects of drowsiness**

Our primary statistical analyses revealed that sulpiride had significant effects on choice accuracy and win-stay behaviour relative to placebo. Separately, we observed a trend suggesting that sulpiride may have altered subjective feelings of drowsiness, but this effect did not reach statistical significance. In light of this trend in the data, we ran a control analysis in which we used linear regression models to investigate whether observed differences in task performance could be explained by drowsiness. Separate models tested task performance (Accuracy or Win-stay behaviour) as a function of Drug, and included Drowsiness as a covariate. Drowsiness was based on the BL-VAS rating made closest to the time each participant commenced the behavioural task.

As in the primary analyses, the main effect of Drug was significant in both models, showing lower Accuracy on sulpiride vs. placebo (∆Acc = -0.04 ± 0.02; t = -2.07; p = .046) and lower Win-stay on sulpiride vs. placebo (∆Win-stay = -0.05 ± 0.02; t = -2.13; p = .04). Neither Accuracy nor Win-stay was significantly associated with Drowsiness (p ≥ .09).

**Controlling for effects of effort variability**

In a control analysis, we used linear regression to investigate whether observed differences in task performance could be explained by changes in effort variability throughout the task. Effort variability was measured for each participant as the standard deviation of observed peak force amplitudes (Effort SD). Each model tested task performance (Accuracy and Win-stay behaviour) as a function of Drug, while controlling for Effort SD as a covariate. Consistent with our primary analyses, the main effect of Drug was significant in both models, showing lower Accuracy on sulpiride vs. placebo (∆Acc = -0.04 ± 0.02; t = -2.14; p = .039) and lower Win-stay on sulpiride vs. placebo (∆Win-stay = -0.05 ± 0.02; t = -2.36; p = .023). Neither Accuracy nor Win-stay was significantly associated with Effort SD (p ≥ .32). These analyses thus confirm that the observed effects of sulpiride on task performance were not associated with changes in effort variability.

**Testing differences between experimental blocks**

In a set of control analyses, we tested for differences in task performance between the first and second experimental blocks. Specifically, we ran a series of mixed effects ANOVAs testing for differences in the dependent variables of Accuracy, Effort Exertion, Win-stay behaviour, and Lose-switch behaviour. For each of these ANOVAs, we examined the effects of Drug (placebo, sulpiride) as a between-subjects factor, Block (first, second) as a within-subjects factor, and the Drug x Block interaction.

All of these ANOVAs replicated the findings described in the primary analyses, including significant main effects of Drug on Accuracy and Win-stay behaviour (∆Acc = 0.04 ± 0.02, F(1,40) = 4.33, p = .044; ∆Win-stay = 0.05 ± 0.02, F(1,40) = 5.15, p = .029; ∆Lose-switch and ∆Effort Exertion, both p ≥ .16). Notably, in none of the ANOVAs on Accuracy, Effort Exertion, and Lose-switch behaviour was the main effect of Block or the Drug x Block interaction significant (p ≥ .36). The only analysis that revealed a trend involving Block was the Win-stay ANOVA, which showed that the Drug x Block interaction was of borderline significance (Drug x Block, F(1,40) = 3.51, p = .068; Block, p = .75), potentially driven by sulpiride producing a greater reduction in Win-stay rate in Block 1 (0.07 ± 0.03) compared to Block 2 (0.04 ± 0.02). Overall, these analyses indicate that there were no significant changes in behaviour between blocks in the first session.

**Testing the effect of sulpiride on choice exploration**

Our primary analysis revealed that sulpiride significantly reduced choice accuracy relative to placebo. In a supplementary analysis, we investigated whether this effect could be explained by increased exploratory decision-making, as opposed to an effect on learning per se. To test this, we examined blocks with unequal contingencies (i.e., P = 0.3 vs. 0.7), and focused our analysis on the latter part of these blocks once participants had finished learning these contingencies. We defined this period as beginning at the inflection point in each group’s learning curve – that is, from the first trial on which accuracy was the same or lower than on the previous trial. We reasoned that, if the effect of sulpiride on accuracy was driven by exploratory behaviour, then the effect should remain prominent in these periods after learning. However, this was not the case – we found no difference in choice accuracy between drug groups once participants had learned the new contingencies (placebo: Acc = 0.78 ± 0.02; sulpiride: Acc = 0.75 ± 0.02; t(40) = 0.93; p = .36; refer to S4 Fig, file: S4_Fig.tif).

**Exploring the interaction between *k* and *φ***

The winning model in the placebo group (M3) raised the possibility that effort improves learning in the most effort-averse individuals. We tested this by fitting a linear regression model predicting mean accuracy in the placebo group as a function of effort aversion (*k*), learning rate asymmetry (*φ*), and their interaction. Although none of these parameters was significant (p ≥ .21), an exploratory simple slopes analysis revealed that the direction of the interaction effect may be consistent with an adaptive role for effort in participants who are most averse to high effort actions (refer to S5 Fig, file: S5_Fig.tif). We explore this further in the simulation analyses presented in the main text.

**Computational models with declining learning rates**

In a supplementary modelling analysis, we considered whether differences in choice behaviour between drug groups could be explained by an effect of sulpiride on learning rates that decline between changes in reward contingencies. To investigate this, we developed three control models (CM1-3) designed to test the prediction that learning rates (and learning rate asymmetries) should be maximal after a switch in stimulus-reward contingencies, and decrease over subsequent trials.

CM1 was similar to M1, except that it allowed for the possibility that signal gain (*γ*) decreases as a function of trial number in the current contingency block. We use *i* to denote within-block trial number, which resets to 0 on the first trial following each contingency change. The rate at which *γ* decreases as a function of *i* is captured by an additional free parameter, *τ*.

|  | *CM1:* | $G\left( t \right)=\gamma-\tau\cdot i(t)$ | (10) |
| --- | --- | --- | --- |

CM2 takes a similar form, except in this case it is not learning rate but learning rate asymmetry (*φ*) that decreases over time. The rate at which *φ* decreases is again captured by a free parameter (*τ*). This model is otherwise identical to M2 from the original model space.

|  | *CM2:* | $G\left( t \right)=\left\{ \begin{aligned} \gamma+(\varphi_{N}-\tau\cdot i\left( t \right)), \delta(t)>0 \\ \gamma-(\varphi_{N}-\tau\cdot i\left( t \right)), \delta\left( t \right)<0 \end{aligned} \right.$ | (11) |
| --- | --- | --- | --- |

Finally, CM3 extends this to the case that learning rate asymmetry is modulated by effort, such that *τ* captures the rate at which the effect of effort decreases over time. This model is otherwise identical to M3 from the original model space.

|  | *CM3:* | $G\left( t \right)=\left\{ \begin{aligned} \gamma+\left( \varphi_{E}-\tau\cdot i\left( t \right) \right)\cdot E_{x}\left( t \right), \delta\left( t \right)>0 \\ \gamma-(\varphi_{E}-\tau\cdot i\left( t \right))\cdot E_{x}\left( t \right), \delta\left( t \right)<0 \end{aligned} \right.$ | (12) |
| --- | --- | --- | --- |

We compared model fits based on the AIC and found that none of these control models was superior to the original winning model in each group. Specifically, choice behaviour was still best explained by M3 in the placebo group (CM1-3 compared to M3, ∆AIC ≥ 19.34), and by M2 in the sulpiride group (CM1-3 compared to M2, ∆AIC ≥ 46.04).

These results thus confirm that learning rates and learning rate asymmetries in this study did not significantly decline over the course of each contingency block. Importantly, this also means that the observed effects of sulpiride on choice behaviour in this study are unlikely to be related to declining learning rates.

**Computational models fit across both drug groups**

Model comparisons showed that the *Effort Reinforcement model* (M3) provided the best fit in the placebo group, and the *Dual Learning Rates model* (M2) provided the best fit in the sulpiride group. In a supplementary analysis, we asked whether these results could be recapitulated by modelling all participants across both drug groups simultaneously. To investigate this, we compared four supplementary models (SM1-4), which tested competing hypotheses about the effect of sulpiride vs. placebo on the interaction between effort and learning. In this framework, we let the signal gain term (*G*) incorporate separate static (effort-insensitive) and dynamic (effort-sensitive) components of learning rate asymmetry. The static component was fixed at 0.5 to reflect the midpoint of the effort range. The dynamic component varied according to the amount of effort exerted on each trial, centred on each participant’s mean effort (∆*E*). The critical feature of these supplementary models was the inclusion of an additional dopamine signalling parameter (*D*), which modelled the functional state of the dopamine D2 receptor.

|  | *SM1-4:* | $G\left( t \right)=\left\{ \begin{aligned} \gamma+\varphi\left( 0.5 + D\cdot\Delta E\left( t \right) \right), \delta\left( t \right)>0 \\ \gamma-\varphi(0.5 + D\cdot\Delta E\left( t \right)), \delta\left( t \right)<0 \end{aligned} \right.$ | (13) |
| --- | --- | --- | --- |

All four supplementary models adopted this general structure, but models differed with respect to the setting of the dopamine signalling parameter *D*. In the first model (*SM1*), we set *D* to match the true drug allocation of participants in the study, where *D = 1* for participants in the placebo group (denoting normal signalling) and *D = 0* for participants in the sulpiride group (denoting D2 receptor blockade). This model stipulates that effort should modulate learning rates on placebo but not on sulpiride, consistent with the results of our original analysis. We then examined how well *SM1* fit the observed data compared to three alternative models (*SM2-4*) in which we applied different *D* parameter settings. In *SM2*, we set *D = 0* for participants in the placebo group and *D = 1* for the sulpiride group (i.e., the reverse allocation compared to *SM1*). In *SM3*, we set *D = 0* for all participants (i.e., effort has no effect on learning rates in either drug group). Finally, in *SM4*, we set *D = 1* for all participants (i.e., effort modulates learning rates in both drug groups).

We found that *SM1* provided the best account of the empirical data. Consistent with our original analyses, this model stipulates that effort modulated learning rates in the placebo group, but not in the sulpiride group. This account provided a superior fit compared to *SM2,* in which the drug group allocations were reversed (∆AIC = 41.58)*,* and compared to *SM3* (∆AIC = 20.61) and *SM4* (∆AIC = 20.97), which stipulated no difference between drug groups. This result thus confirms that a significant effect of sulpiride was to disrupt the effect of effort on learning.
