# Supplementary figures and images for: Dopamine D2-receptor blockade in humans disrupts the effect of effort on learning
Source: PLoS Biol. 2026 Apr 16;24(4):e3003765. doi: 10.1371/journal.pbio.3003765 (PMC13099096; doi:10.1371/journal.pbio.3003765)

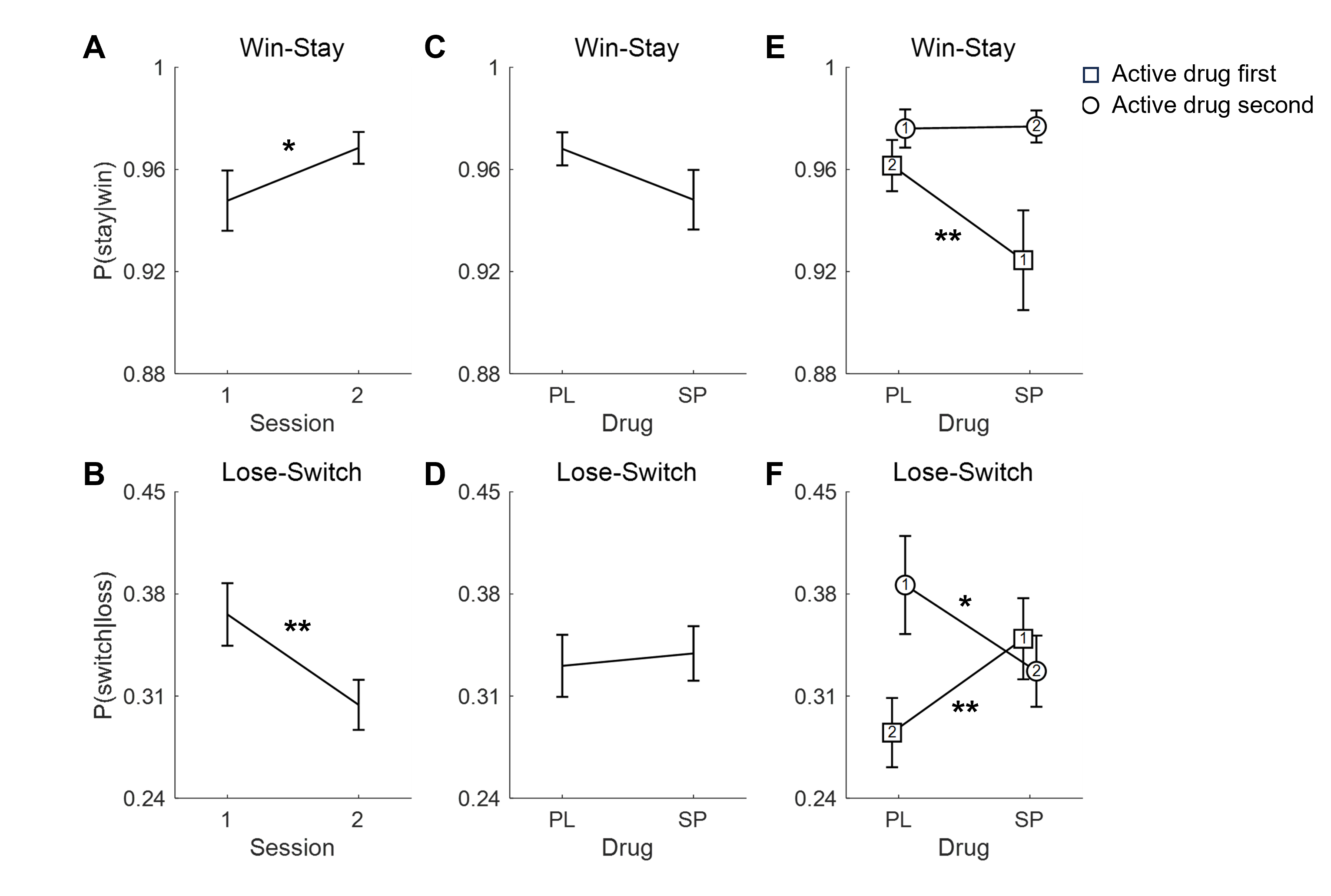

Supplement: S1 Fig — PL, placebo; SP, sulpiride. Total N = 42. Error bars depict the standard error of the mean. Numbers inside markers in panels E and F denote first and second sessions. *p < .05, **p < .01. Underlying data can be found in S1 Data. (TIF) [file pbio.3003765.s003.tif]

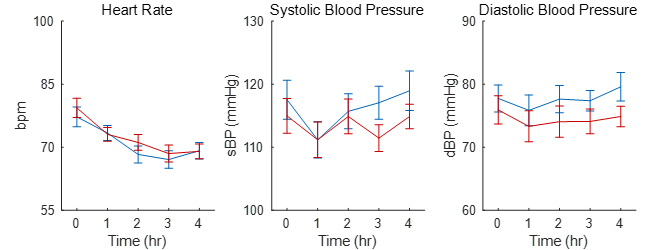

Supplement: S2 Fig — Effects are plotted as a function of time post-ingestion of the sulpiride (red, n = 23) or placebo (blue, n = 19) capsule. Error bars depict the standard error of the mean. bpm, beats per minute; sBP, systolic blood pressure; dBP, diastolic blood pressure. Underlying data can be found in S1 Data. (TIF) [file pbio.3003765.s004.tif]

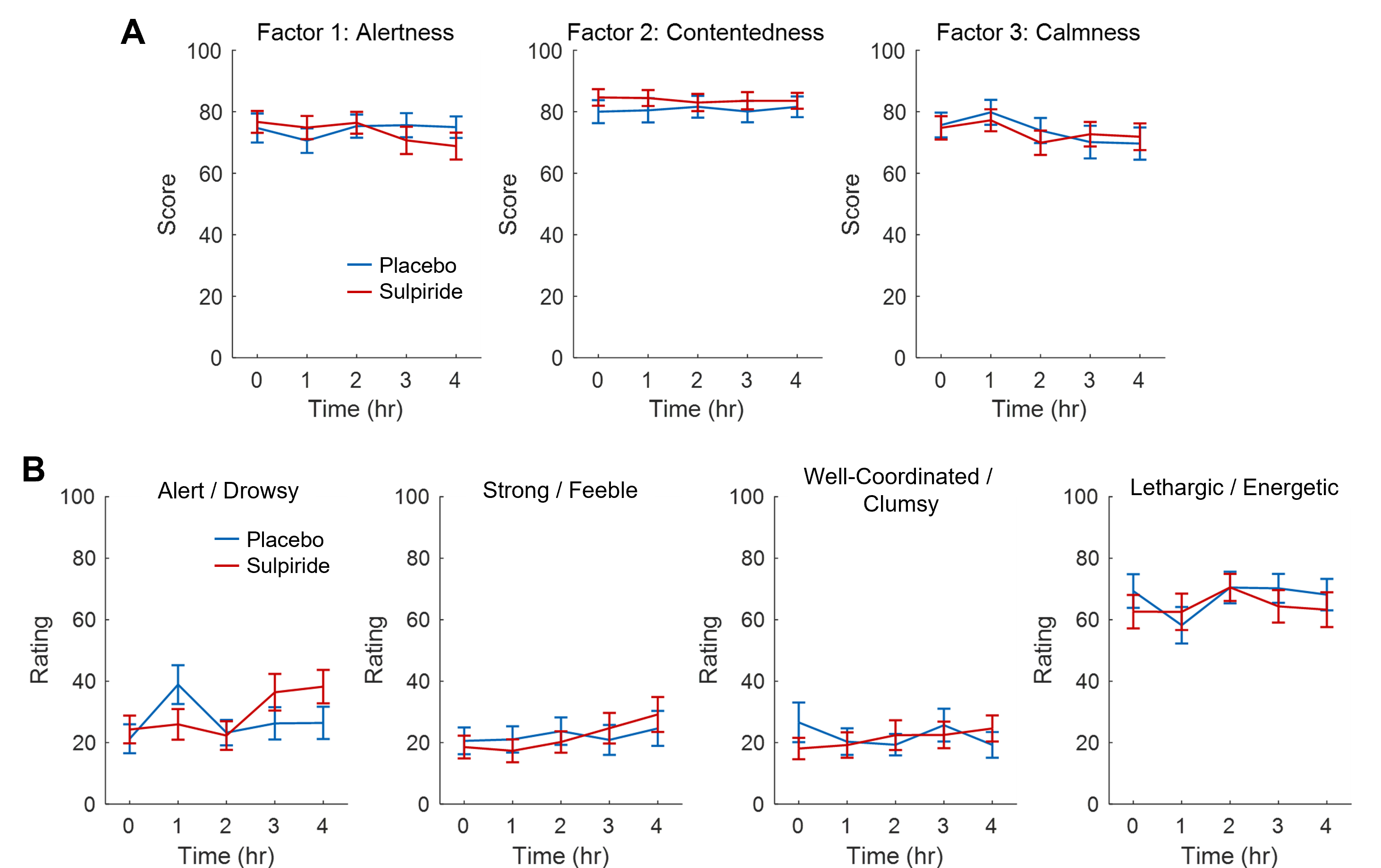

Supplement: S3 Fig — Effects are plotted as a function of time post-ingestion of the sulpiride (red, n = 23) or placebo (blue, n = 19) capsule. (A) Aggregated factor scores. (B) Selected individual scales. Error bars depict the standard error of the mean. Underlying data can be found in S1 Data. (TIF) [file pbio.3003765.s005.tif]

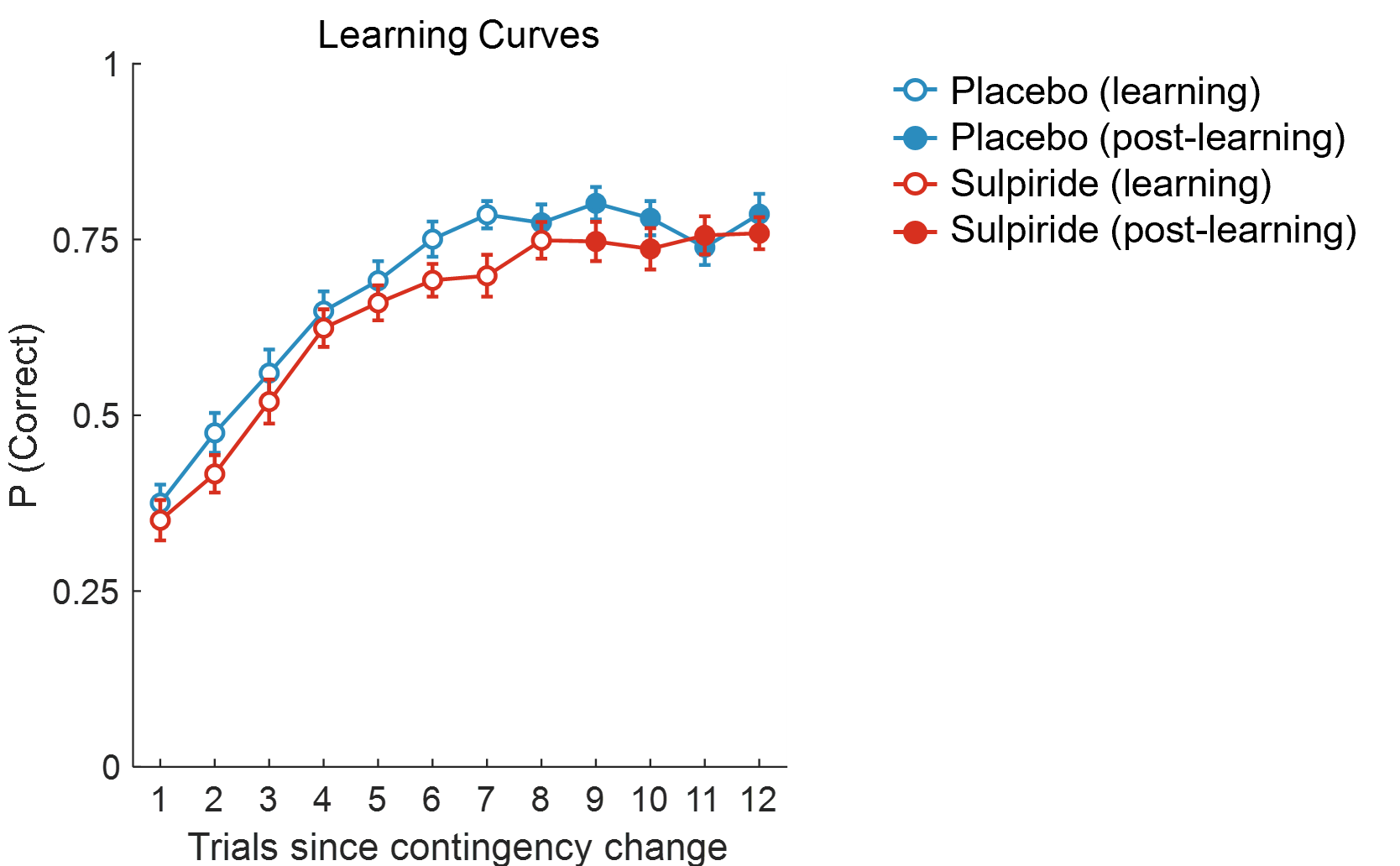

Supplement: S4 Fig — Accuracy (mean ± SEM; y-axis) in the sulpiride (red, n = 23) and placebo (blue, n = 19) groups on each trial since the most recent change in stimulus-reward contingencies (x-axis). Hollow markers depict accuracy during learning, solid markers depict accuracy after contingencies have been fully learned. Underlying data can be found in S1 Data. (TIF) [file pbio.3003765.s006.tif]

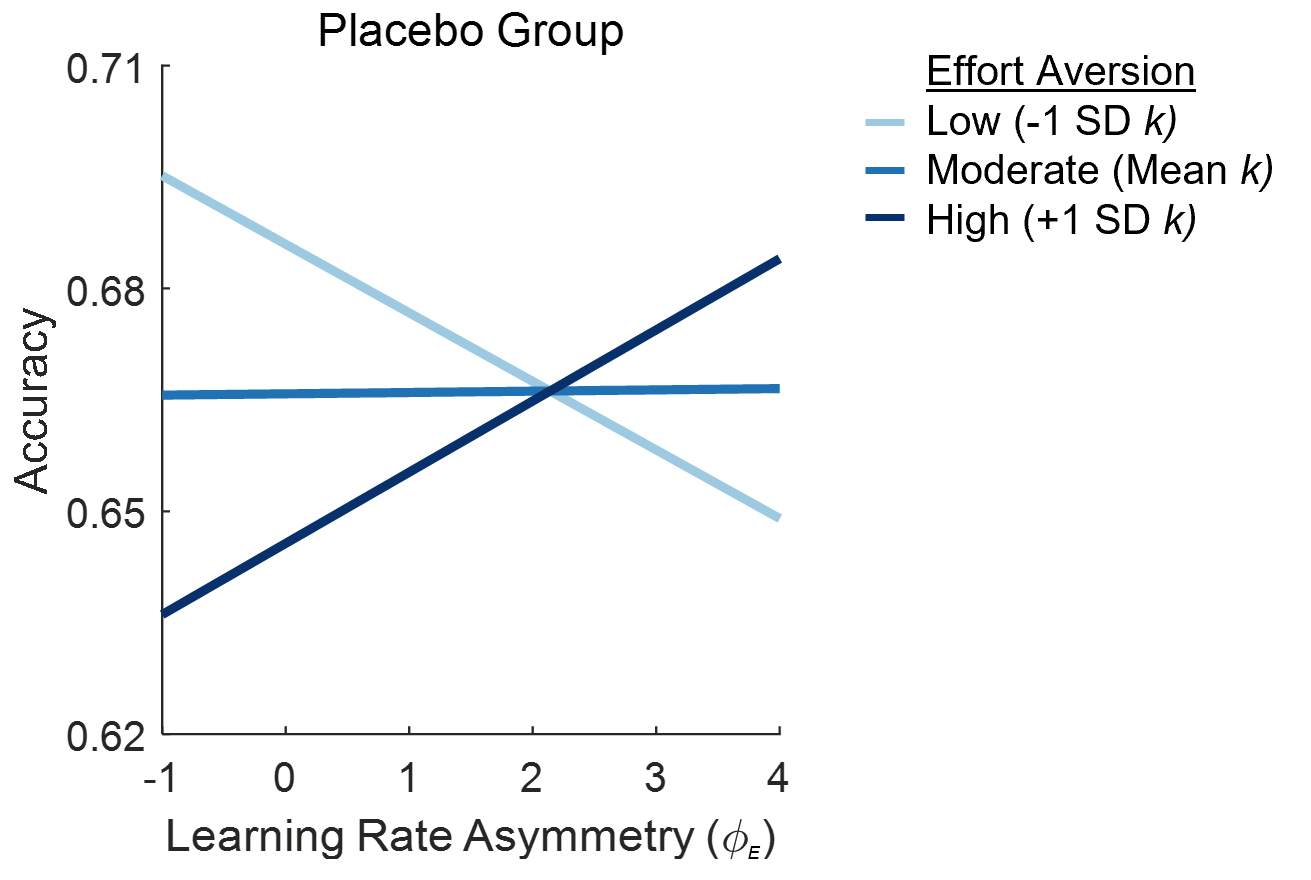

Supplement: S5 Fig — Exploratory simple slopes analysis showing predicted choice accuracy (y-axis) as a function of learning rate asymmetry (φ; x-axis) and effort aversion (k; colors) derived from M3 in the placebo group. (TIF) [file pbio.3003765.s007.tif]
