# Supplementary material for: Dopamine D2-receptor blockade in humans disrupts the effect of effort on learning
Source: PLoS Biol. 2026 Apr 16;24(4):e3003765. doi: 10.1371/journal.pbio.3003765 (PMC13099096; doi:10.1371/journal.pbio.3003765)
Supplement: Attachment — Submitted filename: PLOSBio_ResponseToReviewers.docx [file pbio.3003765.s010.docx]

**Author response to reviewers**

We thank the Editor and Reviewers for their engagement with the work and suggestions for improvement. Below we provide our responses in **black**, with Reviewer comments in **blue**. Sections highlighted in yellow indicate material that has been substantially added or edited in the revised manuscript. Page and line numbers refer to the marked-up version of the revised manuscript.

**Reviewer 1**

Jarvis et al investigated the role of dopamine in the interplay between effort exertion and reinforcement learning. They used sulpiride to block dopaminergic signalling in a between-subject design where participants perform a probabilistic reinforcement learning task where they have to also exert the right amount of effort to obtain reward. Sulpiride impaired choice accuracy and win-stay behaviour. They fit a set of RL models and find that a model that incorporates effort exertion fits best in the placebo group (replicating their previous work), but that this is not the case for the sulpiride group. Further analysis indicates that D2 receptor blockade disrupts the relationship between motivation to exert effort and learning rate, leading to impaired performance in a reinforcement learning task.

Overall, I found the results the authors present interesting and reasonably compelling, though their conclusions are not very straightforward and therefore not the easiest to follow. In addition, there are several aspects of the reporting that are not very clear or are lacking, and including these would make the work a lot more compelling.

We thank the Reviewer for their interest, and their comments in improving the manuscript.

As the authors state, the participants in this study receive a high dose of sulpiride. Whilst this is commendable as it ensures the effects are post-synaptic, I was somewhat surprised there were no reports of side-effects at all. At this dosage, parkinsonian symptoms surely would be likely. Did the researchers only administer the BL-VAS, or did they also include ask questions more targeted to parkinsonian symptoms specifically?

Thank you for raising this issue. We agree that parkinsonian symptoms are possible following ingestion of 800 mg sulpiride, but in this case we observed no such symptoms. We note that this is consistent with previous studies in healthy humans, which also reported no side effects when using the same dose of sulpiride (Takano, Suhara et al. 2006, Eisenegger, Naef et al. 2014).

Other than the BL-VAS, heart rate and blood pressure, we did not take measures specifically targeted to parkinsonian symptoms. However, the four scales of the BL-VAS included in our initial analysis were selected because they were deemed most relevant for detecting possible motor and cognitive effects of the drug. These were scales measuring the degree to which participants felt ‘alert/drowsy’, ‘strong/feeble’, ‘well-coordinated/clumsy’, and ‘lethargic/energetic’. The fact we found only small differences in the trajectory of ‘alert/drowsy’ ratings between drug groups, and no significant differences on the other scales, suggests that parkinsonian symptoms were minimal, if present at all.

For thoroughness, we now include an additional analysis of aggregated scores based on all BL-VAS scales, in keeping with previous studies (Bond and Lader 1974, Chamberlain, Müller et al. 2006, Eisenegger, Naef et al. 2014). Specifically, we used the individual scales to calculate three weighted averages per participant, which measured distinct factors of ‘alertness’, ‘contentedness’, and ‘calmness’, respectively (Bond and Lader 1974). This additional analysis again showed no significant differences between drug groups, which provides further evidence that there were no detectable adverse drug reactions.

We have updated the Results section of the revised manuscript to reflect these changes (page 6, lines 142-144):

There were no effects of sulpiride vs. placebo on subjective reports of feeling strong/feeble, well-coordinated/clumsy, or lethargic/energetic, nor on aggregated factors of alertness, contentedness, and calmness (***Fig. S3***).

We also provide further detail in the Supporting Information (page 36, lines 960-981):

At the start of the testing session and after every hour, participants used (digitised) Bond and Lader visual analogue scales (BL-VAS) to report their current subjective state. Scales measured the degree to which participants felt ‘alert/drowsy’, ‘calm/excited’, ‘strong/feeble’, ‘muzzy/clear-headed’, ‘well-coordinated/clumsy’, ‘lethargic/energetic’, ‘contented/discontented’, ‘troubled/tranquil’, ‘mentally slow/quick-witted’, ‘tense/relaxed’, ‘attentive/dreamy’, ‘incompetent/proficient’, ‘happy/sad’, ‘antagonistic/amicable’, ‘interested/bored’, and ‘withdrawn/gregarious’ (Bond and Lader 1974). In keeping with previous studies, these individual scales were used to calculate weighted averages measuring three distinct factors of ‘alertness’, ‘contentedness’, and ‘calmness’, respectively (Bond and Lader 1974, Chamberlain, Müller et al. 2006, Eisenegger, Naef et al. 2014). In addition, four scales were selected for individual analysis to aid in the detection of any possible motor changes caused by sulpiride. These were scales measuring the degree to which participants felt ‘alert/drowsy’, ‘strong/feeble’, ‘well-coordinated/clumsy’, and ‘lethargic/energetic’.

Separate mixed-effects ANOVAs were run to examine differences in these measures. Each ANOVA included Drug (placebo, sulpiride) as a between-subjects factor, Time (0-4 Hrs) as a within-subjects factor, and their interaction. There were no significant effects of Drug on any of the factor scores (main effect and interactions, all p ≥ .1). The ANOVA on the ‘alert/drowsy’ scale showed a significant Drug x Time interaction effect (F(2.53,101.3) = 3.52, p = .024). This was driven by small differences in the trajectory of this measure (i.e., an early peak in the placebo group and a later peak in the sulpiride group), but there were no significant differences when comparing drug groups at each individual time point (post hoc t-tests at hrs 0-4, all p ≥ .55). There were no other significant effects of Drug on the individual scales (main effect and interactions, all p ≥ .15).

Revised Fig. S3 (page 37):

***Fig S3. Drug effects on subjective feelings.*** Effects are plotted as a function of time post-ingestion of the sulpiride (red) or placebo (blue) capsules. (A) Aggregated factor scores. (B) Selected individual scales.

Relatedly, whilst the authors do not report any difference in drowsiness at any individual time point, they did find a change in trajectory which may be informative. Did the authors try explaining changes in task performance (accuracy and win-stay behaviour) with this subjective measure taken around the time point when the participants performed the task?

Thank you for this suggestion. We have now conducted a post hoc analysis in which we used linear regression models to investigate the effect of the drug on task performance (accuracy and win-stay behaviour) while controlling for drowsiness. These models confirmed that changes in task performance could not be explained by drowsiness.

We have outlined this analysis in the Supporting Information (page 38-39, lines 1002-1014):

**Controlling for effects of drowsiness**

Our primary statistical analyses revealed that sulpiride had significant effects on choice accuracy and win-stay behaviour relative to placebo. Separately, we observed a trend suggesting that sulpiride may have altered subjective feelings of drowsiness, but this effect did not reach statistical significance. In light of this trend in the data, we ran a control analysis in which we used linear regression models to investigate whether observed differences in task performance could be explained by drowsiness. Separate models tested task performance (Accuracy or Win-stay behaviour) as a function of Drug, and included Drowsiness as a covariate. Drowsiness was based on the BL-VAS rating made closest to the time each participant commenced the behavioural task.

As in the primary analyses, the main effect of Drug was significant in both models, showing lower Accuracy on sulpiride vs. placebo (∆Acc = -0.04 ± 0.02; t = -2.07; p = .046) and lower Win-stay on sulpiride vs. placebo (∆Win-stay = -0.05 ± 0.02; t = -2.13; p = .04). Neither Accuracy nor Win-stay was significantly associated with Drowsiness (p ≥ .09).

It is not very clear how the effectiveness of the blinding was assessed, currently just a single p-value is reported with no explanation of what was tested. It would be useful to include the blinding data in a table, and to include a standardised index like the Bang Blinding Index (Bang et al., 2004 *Control Clin Trials*). Were the researchers running the experiment also asked what condition the participant was assigned to, if so, report that data also?

At the conclusion of the study, the researchers who ran the experiment reported that they had been unable to determine the group assignment of the participants. However, these data were not systematically recorded for each individual participant, and thus we cannot formally assess the efficacy of researcher blinding.

As suggested by the Reviewer, we have now assessed participant blinding by calculating the Bang Blinding Index (BBI) for the placebo and sulpiride groups. BBI scores were close to zero in both groups, which is consistent with effective blinding (placebo, BBI = 0 ± 0.17 (mean ± SEM), 95% CI = [-0.33, 0.33]; sulpiride, BBI = 0.26 ± 0.15, 95% CI = [-0.04, 0.56]). As requested, we have also included the blinding data in the manuscript in tabular format.

In addition, we performed a control analysis to confirm that the observed differences in task performance between drug groups (i.e., accuracy and win-stay behaviour) were not related to participant awareness of their assigned drug group. These additional analyses are outlined in the Methods section (page 6, lines 146-154):

At the conclusion of testing, participants were asked whether they believed they had ingested the placebo, the active drug, or if they were unsure (***Tab. S1***). These data were then used to calculate the Bang Blinding Index (BBI) for each drug group (1 = complete lack of blinding; 0 = perfect blinding; -1 = complete attribution of assignment to alternate group; Bang, Ni et al. 2004). Importantly, the BBI in both groups was close to zero, which reflects effective blinding in our study (placebo, BBI = 0 ± 0.17 (mean ± SEM), 95% CI = [-0.33, 0.33]; sulpiride, BBI = 0.26 ± 0.15, 95% CI = [-0.04, 0.56]). In addition, a supplementary analysis demonstrated that behaviour was not significantly related to whether participants were able to correctly guess their assigned drug group (see ***Supporting Information***).

Further details are provided in the Supporting Information (page 38, lines 987-1000):

**Assessment of participant blinding**

| **Assignment** | **Response** |  |  |  |
| --- | --- | --- | --- | --- |
|  | Sulpiride | Placebo | Unsure | Total |
| Sulpiride | 10 | 4 | 9 | 23 |
| Placebo | 5 | 5 | 9 | 19 |
| Total | 15 | 9 | 18 | 42 |

***Table S1. Participant blinding data.* ‘**Assignment’ refers to the true allocation of participants to the sulpiride and placebo groups. ‘Response’ refers to the participants’ belief about their allocated group (‘placebo’, ‘sulpiride’, or ‘unsure’).

As reported in the main text, we assessed blinding efficacy with the Bang Blinding Index. In addition, we confirmed that the significant effect of sulpiride vs. placebo on task performance (i.e., accuracy and win-stay behaviour) was not related to participants’ awareness of their assigned drug group. We ran a two-way ANOVA testing whether Accuracy differed according to: (1) the drug that participants Guessed they had ingested (placebo, sulpiride, unsure); (2) their True Drug group (placebo, sulpiride); and (3) the Guessed Drug × True Drug interaction. In addition, we conducted the analogous ANOVA on Win-stay behaviour instead of Accuracy.

In both ANOVAs, neither the main effect of Guessed Drug, nor its interaction with True Drug, were significant (p ≥ .19). This confirms that learning performance was not affected by participants’ belief of which drug they had consumed.

The authors conclude that sulpiride did not affect motor performance as there was no group difference in mean effort generation. However, could it be that participants within the sulpiride group produced more variable effort?

To address this question, we examined the effect of sulpiride on effort variability, which we measured for each participant as the standard deviation of observed peak force amplitudes (Effort SD). Specifically, we ran an ANOVA testing for differences in Effort SD by Drug group (placebo, sulpiride), Stimulus type (low effort, high effort), and their interaction. Importantly, the main effect of Drug group was not statistically significant. We have added this analysis to the Results section in the revised manuscript (page 13, lines 309-313):

We then ran an equivalent ANOVA testing differences in the standard deviation of peak force across responses (Effort SD). Importantly, the main effect of Drug group was not significant (ΔEffort SD = 0.01 ± 0.01 (mean ± SEM), F(1,40) = 3.36, p = .074; ***Fig. 3C***). The effect of Stimulus in this case was not significant (main and interaction both p ≥ .22; ***Fig. 3C***).

Although the effect of sulpiride on effort variability was not statistically significant, we nevertheless tested whether it could be driving other differences in performance between drug groups. Specifically, we ran a post hoc analysis using the same linear regression approach that we conducted for the analysis on ‘drowsiness’ suggested by the Reviewer. This confirmed that the observed effects of sulpiride on learning performance could not be explained by differences in effort variability. We outline this additional analysis in the Supporting Information (page 39, lines 1015-1025):

**Controlling for effects of effort variability**

In a control analysis, we used linear regression to investigate whether observed differences in task performance could be explained by changes in effort variability throughout the task. Effort variability was measured for each participant as the standard deviation of observed peak force amplitudes (Effort SD). Each model tested task performance (Accuracy or Win-stay behaviour) as a function of Drug, while controlling for Effort SD as a covariate. Consistent with our primary analyses, the main effect of Drug was significant in both models, showing lower Accuracy on sulpiride vs. placebo (∆Acc = -0.04 ± 0.02; t = -2.14; p = .039) and lower Win-stay on sulpiride vs. placebo (∆Win-stay = -0.05 ± 0.02; t = -2.36; p = .023). Neither Accuracy nor Win-stay was significantly associated with Effort SD (p ≥ .32). These analyses thus confirm that the observed effects of sulpiride on task performance were not associated with changes in effort variability.

I am having trouble parsing the statement that 'learning rates are sensitive to effort exertion'. Is this not just a feature of the task, i.e. you can only learn when you exert above threshold effort? It may be that I just didn't understand this, due to lack of understanding of the task.

In this case, the term ‘learning rate’ refers to a specific computational parameter whose function is to scale the effect of the reward prediction error on each trial. In the effort model introduced in the current paper (i.e., M3), the learning rate parameter is sensitive to trial-by-trial effort exertion. Specifically, the model stipulates that the modulation of learning rate is driven, not by the mere fact of succeeding or failing to meet the effort threshold, but by the precise amount of effort exerted on each trial.

The Reviewer’s understanding of the task is correct, in that participants were indeed required to exert above threshold effort in order to select a stimulus and learn from the resulting reward feedback. Critically, however, they were well-practised on this aspect of the task before beginning the experimental blocks. As a result, they very rarely failed to meet the effort threshold: participants missed fewer than 0.7% of trials on average, and this number did not differ significantly between drug groups (p = .15). It is therefore highly unlikely that learning performance was related merely to participants’ capacity to meet the effort threshold. We have added this data to the manuscript to avoid confusion on this aspect of the task.

Revised section of manuscript (page 13, lines 300-303):

We also confirmed that participants were able to successfully meet the required effort thresholds when registering responses in the experimental blocks (< 0.7% of trials missed per participant on average), and found that this did not differ significantly between drug groups (z = -1.46, p = .15).

Figure 3D-F: are the group differences across both effort conditions or is there an interaction? Could the bars be split per condition to make this more clear?

In response to the Reviewer’s question, we have revised our analyses to incorporate an effect of Stimulus type on behaviour. Specifically, for each of the behavioural metrics (Accuracy, Win-stay, Lose-switch), we conducted an ANOVA on the effects of Drug group (placebo, sulpiride), and Stimulus type (low effort, high effort). These analyses reiterated the significant effect of Drug group on Accuracy and Win-stay behaviour, and showed that this effect was across both effort conditions – i.e., the Drug x Stimulus interaction was not significant in either case. Of course, the main effect of Stimulus type was significant in all three analyses, in keeping with the phenomenon of effort aversion – this replicates our previous work (Jarvis, Stevenson et al. 2022). We have reported these additional analyses in the main manuscript (page 14-15, lines 336-359):

We used a two-way ANOVA to compare Accuracy between Drug groups (placebo, sulpiride) as a function of the Stimulus type (low effort, high effort). This revealed that, across both stimulus types, sulpiride reduced overall choice accuracy compared to placebo (Drug, ∆Accuracy = 0.04 ± 0.02, t(40) = 2.1, p = .042; Stimulus main effect and interaction, p ≥ .15; ***Fig. 3E***).

We next ran analogous ANOVAs to investigate whether sulpiride altered the tendency to make Win-stay and Lose-switch choices, as more specific measures of reward-guided behaviour. We found a significant main effect of Drug in the Win-stay analysis, indicating that the sulpiride group was less likely than the placebo group to repeat rewarded choices (∆Win-stay = 0.05 ± 0.02, t(40) = 2.27, p = .029; ***Fig. 3F***). This analysis also revealed a main effect of Stimulus type, which reflected that, across both groups, participants were less likely to repeat rewarded high effort choices than rewarded low effort choices (∆Win-stay = -0.02 ± 0.01, t(40) = -2.75, p = .009; ***Fig. 3F***). Notably, the Drug × Stimulus interaction was not significant (p = .89).

In the Lose-switch analysis, we found no significant difference between Drug groups (p = .4; ***Fig. 3G***). However, we again found a significant main effect of Stimulus type, which in this case indicated that participants were more likely to switch stimulus following non-rewarded high effort choices than non-rewarded low effort choices (∆Lose-switch = 0.03 ± 0.01, t(40) = 2.9, p = .006; ***Fig. 3G***). Together with the Win-stay analysis, these effects of Stimulus type are indicative of effort aversion, such that participants were in general less likely to repeat high effort choices than low effort choices. Again, the Drug × Stimulus interaction effect was not significant, providing further evidence that sulpiride did not alter effort aversion in this study (p = .75).

In sum, these analyses reveal that overall reward-related performance was lower on sulpiride than placebo, and that this was associated with changes in win-stay, but not necessarily lose-switch, behaviour.

With regards to Fig. 3E-G, these originally showed group differences collapsed across both effort conditions. As suggested by the Reviewer, we have now split the bars by effort condition to accompany the revised analysis incorporating stimulus type (low effort vs. high effort) (page 14):

***Fig 3. Motor and learning performance in the sulpiride and placebo groups.*** (A-C) Motor performance did not differ significantly between drug groups with respect to (A) MVCs, (B) mean effort exerted during the experimental blocks, or (C) standard deviation of effort during the experimental blocks. Dotted lines in (B) indicate minimum effort thresholds for the low and high effort stimulus, respectively. (D) Sulpiride did not affect preferences for low vs. high effort actions compared to placebo. (E-G) Learning performance differed between drug groups. Relative to placebo, participants on sulpiride exhibited (E) significantly lower choice accuracy (p = .044), and (F) were less likely to repeat rewarded choices (p = .005), but (G) were no different in their tendency to switch stimulus following unrewarded choices (p = .4). In addition, across both drug groups, participants were less likely to repeat high effort choices than low effort choices, consistent with effort aversion (F-G). Statistical significance marks are shown for differences between drug groups. *p < .05; **p < .01; PL = placebo; SP = sulpiride.

The authors show that M2 fits the sulpiride group data better than M3, which has a better fit in placebo. As the model fits were done on the two groups separately rather than including a factor that accounts for the treatment group, this makes me wonder whether there any difference in how well the models fit the data of the two groups, especially since the differences between model fits (how much better M2/3 do compared to M1) varies quite drastically between the two treatment groups. If I eyeball it from Figure 4C and F it looks to me like the absolute model fit for the placebo data looks a bit better.

We are hesitant to directly compare absolute model fits *between* the two drug groups, because these are derived from different winning models (i.e., M2 vs. M3) and different sample sizes (i.e., n = 23 vs. 19). For example, given that model fits are based on summed AIC scores in each group, the relative differences pointed out by the Reviewer (i.e., M2/3 vs. M1 shown in Fig. 4) also reflect sample size differences rather than differences in predictive accuracy alone.

We nonetheless appreciate the Reviewer’s comment that our current models do not include a factor accounting for drug group. We have now addressed this in a supplementary analysis in which we fit a unified model to all participants across both drug groups, which allowed us to test competing hypotheses about the effect of sulpiride vs. placebo based on a single model. This analysis confirmed our original results. We include this analysis in the Supporting Information (page 42-43, lines 1101-1130):

**Computational models fit across both drug groups**

Model comparisons showed that the *Effort Reinforcement model* (M3) provided the best fit in the placebo group, and the *Dual Learning Rates model* (M2) provided the best fit in the sulpiride group. In a supplementary analysis, we investigated whether these results could be recapitulated by modelling all participants across both drug groups simultaneously. To investigate this, we compared four supplementary models (SM1-4), which tested competing hypotheses about the effect of sulpiride vs. placebo on the interaction between effort and learning. In this framework, we let the signal gain term (*G*) incorporate separate static (effort-insensitive) and dynamic (effort-sensitive) components of learning rate asymmetry. The static component was fixed at 0.5 to reflect the midpoint of the effort range. The dynamic component varied according to the amount of effort exerted on each trial, centred on each participant’s mean effort (∆*E*). The critical feature of these supplementary models was the inclusion of an additional dopamine signalling parameter (*D*), which modelled the functional state of the dopamine D2 receptor.

|  | *SM1-4:* | $G\left( t \right)=\left\{ \begin{aligned} \gamma+\varphi\left( 0.5 + D\cdot\Delta E\left( t \right) \right), \delta\left( t \right)>0 \\ \gamma-\varphi(0.5 + D\cdot\Delta E\left( t \right)), \delta\left( t \right)<0 \end{aligned} \right.$ | (10) |
| --- | --- | --- | --- |

All four supplementary models adopted this general structure, but models differed with respect to the setting of the dopamine signalling parameter *D*. In the first model (*SM1*), we set *D* to match the true drug allocation of participants in the study, where *D = 1* for participants in the placebo group (denoting normal signalling) and *D = 0* for participants in the sulpiride group (denoting D2 receptor blockade). This model stipulates that effort should modulate learning rates on placebo but not on sulpiride, consistent with the results of our original analysis. We then examined how well *SM1* fit the observed data compared to three alternative models (*SM2-4*) in which we applied different *D* parameter settings. In *SM2*, we set *D = 0* for participants in the placebo group and *D = 1* for the sulpiride group (i.e., the reverse allocation compared to *SM1*). In *SM3*, we set *D = 0* for all participants (i.e., effort has no effect on learning rates in either drug group). Finally, in *SM4*, we set *D = 1* for all participants (i.e., effort modulates learning rates in both drug groups).

We found that *SM1* provided the best account of the empirical data. Consistent with our original analyses, this model stipulates that effort modulates learning rates in the placebo group, but not in the sulpiride group. This account provided a superior fit compared to *SM2,* in which the drug group allocations were reversed (∆AIC = 41.58)*,* and compared to *SM3* (∆AIC = 20.61) and *SM4* (∆AIC = 20.97), which stipulated no difference between drug groups. This result thus confirms that a significant effect of sulpiride was to disrupt the effect of effort on learning.

Minor

The study was set up originally as a within-person experiment, but session 2 data was not further analysed due to order effects. I would still be interested to briefly hear how the authors interpret the session 1 vs session 2 effects of sulpiride, rather than just a description of the effects which is given at the moment.

We have updated the manuscript to offer a tentative interpretation of the observed session effects, but we emphasise that this interpretation remains speculative due to likely confounding (page 34, lines 910-917):

Together, these interaction effects indicate that the effect of sulpiride was confounded by the order in which it was administered. When sulpiride was taken first, it appeared to increase lose-switch behaviour and reduce win-stay behaviour relative to placebo. However, when sulpiride was taken second, it instead appeared to reduce lose-switch behaviour while not significantly altering win-stay. These session effects on sulpiride are broadly consistent with previous work showing that the role of dopamine on reward learning can vary across different task contexts (Shiner, Seymour et al. 2012). Interpreting these longer-term order effects are further complicated by the role of dopamine in consolidating previous motor skills (Sommer, Costa et al. 2014). Given that our primary aim was to isolate the effect of effort on trial-by-trial reinforcement learning, we analysed our data with a between-subjects design, using data from the first session only (as per van Nuland, Helmich et al. 2020).

In addition, we have updated ***Fig. S1*** to show these effects more clearly by indicating significant session effects, and denoting the session number corresponding to each data point (page 35):

***Fig S1. Order effects on behaviour.*** PL, placebo; SP, sulpiride. Numbers inside markers in panels E and F denote first and second sessions. *p < .05, **p <.01.

Somewhat related to the session effects, were there any differences between the two blocks in the first session, or an effect of trial number of effort exertion/reinforcement learning performance?

Thank you for this question. Our computational modelling analysis is designed to account for trial-by-trial variation in behaviour within each block, as all models were fit to the empirical choice data at the level of single trials. For example, the *Effort Reinforcement* model (M3) takes the precise amount of effort exerted on each trial as an input, which accounts for any changes in effort that may have occurred over the course of a single block.

However, our existing analysis did not specifically test for differences at the level of blocks in the first session. We have now examined this by running a series of mixed effects ANOVAs testing for differences in task performance between blocks on the dependent variables of Accuracy, Effort exertion, Win-stay behaviour, and Lose-switch behaviour. These additional analyses confirmed that there were no significant changes in behaviour between blocks in the first session. We include these additional analyses in the Supporting Information (page 39, lines 1026-1042):

**Testing differences between experimental blocks**

In a set of control analyses, we tested for differences in task performance between the first and second experimental blocks. Specifically, we ran a series of mixed effects ANOVAs testing for differences in the dependent variables of Accuracy, Effort exertion, Win-stay behaviour, and Lose-switch behaviour. For each of these ANOVAs, we examined the effects of Drug (placebo, sulpiride) as a between-subjects factor, Block (first, second) as a within-subjects factor, and the Drug x Block interaction.

All of these ANOVAs replicated the findings described in the primary analyses, including significant main effects of Drug on Accuracy and Win-stay behaviour (∆Acc = 0.04 ± 0.02, F(1,40) = 4.33, p = .044; ∆Win-stay = 0.05 ± 0.02, F(1,40) = 5.15, p = .029; ∆Lose-switch and ∆Effort exertion, both p ≥ .16). Notably, in none of the ANOVAs on Accuracy, Effort Exertion, and Lose-Switch behaviour was the main effect of Block or the Drug x Block interaction significant (p ≥ .36). The only analysis that revealed a trend involving Block was the Win-stay ANOVA, which showed that the Drug x Block interaction was of borderline significance (Drug x Block, F(1,40) = 3.51, p = .068; Block, p = .75), potentially driven by sulpiride producing a greater reduction in Win-stay rate in Block 1 (0.07 ± 0.03) compared to Block 2 (0.04 ± 0.02).

Overall, therefore, these analyses indicate that there were no significant changes in behaviour between blocks in the first session.

Figure 4C/F: please specify if this is single subject data, the labels ('Placebo group') are making this a bit confusing

The plot shows group-level data. We apologise for omitting this information – we now clarify this in the figure caption (page 17, lines 399-404):

This model provided a superior fit to models M1 and M2 based on AIC scores (A) and Akaike weights (B)~~, and provided an accurate account of observed choices made on placebo (C)~~. (C) M3 also provided an accurate account of observed choices on a trial-by-trial basis. The black line shows the proportion of participants on each trial selecting the low effort stimulus (with standard error in grey), and the blue line shows the mean choice probabilities derived from M3.

Figure 3C-F: can the authors specify whether the bar graphs include all trials or just the 70/30 ones?

Bar graphs depicting accuracy (panel E) are based on trials with reward contingencies P = 0.7 vs. 0.3, as these are trials on which there is one objectively correct response. Other bar graphs are based on all trials. We now state this in the figure caption in the revised manuscript (page 14, line 329-330):

Accuracy in panel E is based on trials with reward contingencies of P = 0.7 vs. 0.3.

line 381: "One possible interpretation of this result is that the dopamine D2-receptor supports" this section focusses on the placebo group analysis, so this sentence seems a bit out of place here

Thank you for pointing this out. We have updated the wording in the revised manuscript to avoid making a claim about receptor function in this section (page 17, lines 424-425):

One possible interpretation of this result is that ~~the dopamine D2-receptor supports an~~ effort plays an adaptive role by facilitating learning in those who are averse to exerting it in the first place.

Figure 5C caption: M3 should be M2, presumably

We have corrected this in the revised manuscript (page 19, line 458).

We thank the Reviewer for their helpful comments and suggestions.

**Reviewer 2**

Here, Jarvis et al. investigated whether effort exerted when making a choice affects learning through dopamine signalling. They used a previously developed two-armed bandit task with probabilistically rewarded options that changed in blocks. Choice selection required exerting either a high or low amount of effort (through squeezing handheld dynamometers), indicated by previously learned stimuli. The authors manipulated dopamine signalling in a subset of participants through systemic blockade of D2 receptors, and found that under such conditions choice behavior was best explained by a model that updated learning rate independently of effort exerted. This was in contrast to the placebo group, for which further simulations revealed that an intact relationship between learning rate and effort might be ecologically adaptive, enabling the maintenance of high levels of accuracy even in agents averse to effort.

This work is a clever, mechanistic extension to the lab's previous findings (Jarvis et al 2022), which itself provided a neat computational unification of two disparate theories of dopamine function: as either shaping effort-based decisions, or as a teaching signal in reinforcement learning. The effects of D2R blockade are relatively robust and the authors perform a number of control analyses to confirm that the effects observed are specific to the interaction between effort and learning rate (or lack thereof). The manuscript is well written and the figures are clear and easy to interpret. Nevertheless, I have some suggestions that are largely aimed at strengthening an already strong set of results, largely centered on deeper analysis of participant behavior.

We thank the Reviewer for their positive feedback, and their comments to improve the manuscript.

Major comments:

1. I am unsure as to whether D2R blockade affects learning per se. It did indeed reduce performance in the task by making participants less likely to choose an option that had just been rewarded. This is not necessarily a learning effect, but might instead be a result of increased exploration (indeed, inverse temperature is lower in this group as shown in fig 5). However, a key element of the behavioral paradigm used is that the probability of reward associated with each option varies independently and across blocks within a session. The effects of this manipulation are not explored anywhere in the paper, but might serve to distinguish slow learning (after block changes)

from persistent exploration. More fundamentally, learning rate - and thus presumably learning rate asymmetry - should be greater around times of block switches. Is this effect seen in the placebo group, and is it disrupted in the sulpiride group - are they slower to learn the new contingencies?

Thank you for this very interesting question. We acknowledge that it can be difficult to distinguish differences in learning from differences in choice exploration, particularly in a learning paradigm like ours in which stimulus-reward contingencies change frequently (i.e., every 12 trials). This design was chosen to promote ongoing learning throughout the experiment, but it also limits the number of decisions that participants make based on fully learned contingencies, which is when exploratory behaviour is easier to distinguish.

We have nonetheless conducted additional analyses in line with the Reviewer’s suggestion to investigate the effect of sulpiride on exploratory behaviour. First, we compared choice behaviour in the latter part of each contingency block, after participants had finished learning the new stimulus-reward contingencies. We reasoned that, if the effect of sulpiride on accuracy was driven by a change in exploratory behaviour, then this effect should remain prominent in periods of minimal learning. However, this was not the case – we found no difference in choice accuracy between drug groups once participants had learned the new contingencies. We outline this analysis in the Supporting Information (page 39, lines 1043-1054):

**Testing the effect of sulpiride on choice exploration**

Our primary analysis revealed that sulpiride significantly reduced choice accuracy relative to placebo. In a supplementary analysis, we investigated whether this effect could be explained by increased exploratory decision-making, as opposed to an effect on learning per se. To test this, we examined blocks with unequal contingencies (i.e., P = 0.3 vs. 0.7), and focused our analysis on the latter part of these blocks once participants had finished learning these contingencies. We defined this period as beginning at the inflection point in each group’s learning curve – that is, from the first trial on which accuracy was the same or lower than on the previous trial (***Fig. S4***). We reasoned that, if the effect of sulpiride on accuracy was driven by exploratory behaviour, then the effect should remain prominent in these periods after learning. However, this was not the case – we found no difference in choice accuracy between drug groups once participants had learned the new contingencies (placebo: Acc = 0.78 ± 0.02; sulpiride: Acc = 0.75 ± 0.02; t(40) = 0.93; p = .36; ***Fig. S4***).

New figure in Supporting Information (page 40):

***Fig S4. Learning curves averaged over contingency blocks.*** Mean accuracy (y-axis) ± SEM in the placebo (blue) and sulpiride (red) groups on each trial since the most recent change in stimulus-reward contingencies (x-axis). Hollow markers depict accuracy during learning, solid markers depict accuracy after contingencies have been fully learned.

Next, we ran a supplementary modelling analysis to test the Reviewer’s intuition that learning rates (and learning rate asymmetries) should be maximal at the time of contingency block changes, and decline over subsequent trials. In both drug groups, we found that our original models provided a superior fit compared to these supplementary models. The observed effect of sulpiride on choice accuracy is therefore unlikely to be related to changes in learning rate over time. We outline this analysis in the Supporting Information (pages 41-42, lines 1076-1099):

**Computational models with declining learning rates**

In a supplementary modelling analysis, we considered whether differences in choice behaviour between drug groups could be explained by an effect of sulpiride on learning rates that decline between contingency changes. To investigate this, we developed three control models (C1-3) designed to test the prediction that learning rates (and learning rate asymmetries) should be maximal after a switch in stimulus-reward contingencies, and decrease over subsequent trials.

C1 was similar to M1, except that it allowed for the possibility that signal gain (*γ*) decreases as a function of trial number in the current contingency block. We use *i* to denote within-block trial number, which resets to 0 on the first trial following each contingency change. The rate at which *γ* decreases as a function of *i* is captured by an additional free parameter, *τ*.

|  | *C1:* | $G\left( t \right)=\gamma-\tau\cdot i(t)$ | (10) |
| --- | --- | --- | --- |

C2 takes a similar form, except in this case it is not learning rate but learning rate asymmetry (*φ*) that decreases over time. The rate at which *φ* decreases is again captured by a free parameter (*τ*). This model is otherwise identical to M2 from the original model space.

|  | *C2:* | $G\left( t \right)=\left\{ \begin{aligned} \gamma+(\varphi_{N}-\tau\cdot i\left( t \right)), \delta(t)>0 \\ \gamma-(\varphi_{N}-\tau\cdot i\left( t \right)), \delta\left( t \right)<0 \end{aligned} \right.$ | (11) |
| --- | --- | --- | --- |

Finally, C3 extends this to the case that learning rate asymmetry is modulated by effort, such that *τ* captures the rate at which the effect of effort decreases over time. This model is otherwise identical to M3 from the original model space.

|  | *C3:* | $G\left( t \right)=\left\{ \begin{aligned} \gamma+\left( \varphi_{E}-\tau\cdot i\left( t \right) \right)\cdot E_{x}\left( t \right), \delta\left( t \right)>0 \\ \gamma-(\varphi_{E}-\tau\cdot i\left( t \right))\cdot E_{x}\left( t \right), \delta\left( t \right)<0 \end{aligned} \right.$ | (12) |
| --- | --- | --- | --- |

We compared model fits based on the AIC and found that none of these control models was superior to the original winning model in each group. Specifically, choice behaviour was still best explained by M3 in the placebo group (∆AIC ≥ 19.34 compared to C1-3), and by M2 in the sulpiride group (∆AIC ≥ 46.04 compared to C1-3).

These results thus confirm that learning rates and learning rate asymmetries in this study did not significantly reduce over the course of each contingency block. Importantly, this also means that the observed effects of sulpiride on choice behaviour in this study are unlikely to be related to declining learning rates.

In sum, these results are broadly consistent with a primary effect of sulpiride on learning – an effect we investigate further in our main modelling analysis and simulations. The additional analyses reported above provide useful clarification, and we have therefore added them to the Supporting Information in the revised manuscript.

2. Figure 6 exploits a simulation approach to illustrate the effects of a range of learning rate asymmetries and degrees of effort aversion on performance in the task. I appreciate that being able to do many runs of a simulation allows for greater statistical power and the ability to systematically explore the effects of a range of parameter values (inspired by the range of those from participants). But I suggest that the authors could go one step further and exploit the individual variability in parameter fits - which is large in both drug and placebo groups - to plot these same metrics but for real, not just simulated data. Although the authors do show a convincing correlation between learning rate asymmetry and effort aversion in the placebo group that is broken by D2R blockade, whether this directly has an effect on accuracy at an individual level is not currently demonstrated.

Thank you for this suggestion. We agree that individual variability in the parameters can sometimes assist in investigating their functional roles. In this case, however, the variability across all four parameters in the model makes interpretation more challenging. For example, it can be particularly difficult to isolate the effects of individual parameters if the observable behaviour arises from a complex interaction between them. One strength of the simulation approach therefore is that it enabled us not only to systematically vary the parameters of interest (i.e., *k* and *φ*), but also to hold other parameters fixed at plausible values (i.e., *γ* and *β*).

In addition, the strong positive correlation between *k* and *φ* in the placebo group implied that we were relatively underpowered to investigate participants with lower *k* and higher *φ,* and vice versa. A further strength of the simulation approach therefore is that it enabled us to sample systematically from those less populated parts of the parameter space, which were of particular relevance to our hypothesis regarding the interaction between *k* and *φ*.

For these reasons, we have opted to retain the simulation approach as our definitive interpretation of the models. For thoroughness, however, we now also report a linear regression that we ran on the empirical parameter estimates in the placebo group, along with a plot of the simple slopes analysis breaking down the (null) interaction effect between *k* and *φ* (Results, page 17, lines 424-430):

One possible interpretation of this result is that ~~the dopamine D2-receptor supports an~~ effort plays an adaptive role by facilitating learning in those who are averse to exerting it in the first place. To test this possibility, we first fit a linear regression model predicting mean accuracy in the placebo group as a function of effort aversion (*k*), learning rate asymmetry (*φ*), and their interaction. Although none of these parameters was significant (p ≥ .21), the direction of the interaction effect would be consistent with an adaptive role for effort in participants most averse to investing it (***Fig S5***). To investigate this more thoroughly, we ran a simulation analysis testing the predictions of model M3 in four groups of *n* = 100 simulated agents (***Fig. 6A-B***).

New figure in Supporting Information (page 41):

***Fig S5. Interaction between k and φ.*** Exploratory simple slopes analysis showing predicted choice accuracy (y-axis) as a function of learning rate asymmetry (φ; x-axis) and effort aversion (*k*; colours) derived from M3 in the placebo group.

3. I understand that as this study was conducted in humans, there is a limit to the precision of the physiological manipulations that can be performed. However, some of the discussion on the role of D2 receptors could be refined to be more consistent with canonical views about their functional attributes. For example, around line 490 there is discussion around transient, effort-induced dopamine signals acting on post-synaptic D2-receptors. However, it is well established that postsynaptic D2Rs are more sensitive to tonic dopamine due to their high baseline affinity, while D2R autoreceptors (expressed presynaptically) are more sensitive to phasic release and can shape dopamine release via a feedback loop. Indeed, there is also work showing deletion of D2 autoreceptors slows reversal learning (<http://dx.doi.org/10.1523/ENEURO.0229-17.2018>). There is other work showing that postsynaptic D2Rs can also in fact be sensitive to phasic dopamine
   (<https://doi.org/10.1016/j.neuron.2014.08.058>) but again, my suggestion considering space and scope constraints is to at least present the canonical view (which is not made clear in the current text). As an aside, I agree with the authors that the tonic/phasic dichotomy is likely more complex than previously appreciated, but it still might hold value strictly in the context of receptor subtype activation which is of course pertinent to the current work. Finally, there is a similar issue in the introduction - beginning line 64, the first mention of D2 receptors is in the context of exerting effort. It is not that I disagree with the citations listed, but my suggestion would be to at least frame them as counter to the canonical model of D2R activation inhibiting action (as Calabresi et al is centered on).

Thank you for this feedback – we appreciate the chance to refine these aspects of the manuscript. We agree with the Reviewer about the paper’s Introduction, and have now updated this section to more clearly present the canonical view of D2Rs in motor inhibition (page 3, lines 63-66):

~~The dopamine D2-receptor plays a particularly important role in motivating and maintaining the exertion of effort.~~ Traditional models of basal ganglia function stipulate that D2 receptors are primarily involved in movement inhibition (Alexander and Crutcher 1990, DeLong 1990), but more recent work has challenged this view. For example, D2 signalling is also linked to increased motor activation (Calabresi, Picconi et al. 2014, Soares-Cunha, Coimbra et al. 2016), and blockade of D2-receptors can heighten the aversiveness of effort (Erfanian Abdoust, Froböse et al. 2024).

We have also amended the subsequent paragraph to include the Linden et al. 2018 paper suggested by the Reviewer showing that deletion of D2Rs slows reversal learning in mice (page 4, lines 79-80):

Disrupting dopaminergic signalling has been shown to impede reward-based learning (Swainson, Rogers et al. 2000, Jocham, Klein et al. 2014, Janssen, Sescousse et al. 2015, Linden, James et al. 2018), with D2-receptor blockade in humans appearing to modulate the relative efficiency with which individuals learn from rewards and punishments (van der Schaaf, van Schouwenburg et al. 2014, Janssen, Sescousse et al. 2015).

With regards to the Reviewer’s comment that “postsynaptic D2Rs are more sensitive to tonic dopamine due to their high baseline affinity, while D2R autoreceptors (expressed presynaptically) are more sensitive to phasic release”, our understanding is the opposite – that presynaptic D2R autoreceptors have higher baseline affinity and are sensitive to tonic dopamine, whereas postsynaptic D2Rs have lower affinity and are activated by phasic bursts (Carlsson 1975, Grace 1991, Elsworth and Roth 1996, Emilien, Maloteaux et al. 1999, Frank and O'Reilly 2006, Ford 2014). We therefore feel that our statement that “transient, effort-induced dopamine signals may act on post-synaptic D2-receptors” is appropriate.

In this section, we now cite the Marcott et al. 2014 paper suggested by the Reviewer as evidence that phasic dopamine signals activate postsynaptic D2Rs. Of course, our suggestion that effort plays a role in this process remains speculative (albeit one consistent with our computational results), and we retain our note of caution in the subsequent sentence that neurophysiological studies are required to confirm this interpretation (Discussion, page 22, lines 538-541):

This result presents the possibility that transient, effort-induced dopamine signals may act on post-synaptic D2-receptors (Marcott, Mamaligas et al. 2014) to modulate phasic learning rates, and that this effect may be disrupted by D2 antagonism. However, the precise neurophysiological correlate of our effect will need to be determined in future studies.

4. This is more of an exploratory question, but I am curious as to what the authors think the limits to this effort effect on learning are? Here the participants exert a (relative to them) small amount of effort even when choosing the high effort option, such that they are always able to exert the effort required (as indicated in fig 3b, although it might be good to mention this explicitly somewhere). This is sensible to avoid confounding effects of risk. But assuming that the main results of this paper generalise to real-world scenarios, do the authors think that the effects of effort on learning continue to follow this same functional form? For example, if an individual exerts a large amount of effort and doesn't get reward, the model here suggests that, if they are very effort averse and thus have a highly asymmetrical learning rate (considering 5B), they will in fact not learn from this lack of reward and are more likely to choose to exert effort again.

Thank you for this excellent question. In principle, the model can accommodate the full range of effort, as the sigmoidal form ensures that learning rates will be constrained to the range [0, 1]. In practice, however, it might be the case that exerting a very high level of effort is encoded as an aversive outcome in of itself – one that may shape the agent’s behaviour to a greater extent than any interaction between effort and reward learning can do.

Regarding the Reviewer’s specific example (i.e., a highly effort-averse individual exerting a large amount of effort but failing to obtain a reward), an important consideration is that, according to the model, such an individual will remain highly averse to effort in absolute terms. In other words, although it might seem counterintuitive that they should be more likely to exert effort again, such an individual might still be less motivated to exert effort than someone wholly less averse to effort in the first place.

Prompted by these considerations, we have added a paragraph to the revised Discussion that considers whether the observed effect of effort might hold in other contexts (page 23, lines 577-589):

An intriguing question is whether the observed effects of effort should hold in contexts other than reward-based learning. For example, recent work has proposed that choice behaviour is shaped not only by rewards, but by the formation of habits via direct strengthening of recently taken actions (Miller, Shenhav et al. 2019, Bennett, Niv et al. 2021, Collins 2025). It seems plausible that the dopaminergic mechanisms underpinning habit formation might also be sensitive to the amount of effort with which these actions are executed (Calabresi, Picconi et al. 2007, Matsuda, Furuta et al. 2009, Greenstreet, Vergara et al. 2025), but to our knowledge this has not been directly tested. It is also unclear whether our results should generalise to learning from punishment – that is, outcomes that are not only worse than expected, but wholly detrimental to the decision-maker. Given the complex role of dopamine (and other systems) in punishment learning (Oleson, Gentry et al. 2012, de Jong, Afjei et al. 2019, Lopez and Lerner 2025), it is difficult to predict the specific modulatory effects of effort in this domain. Our results thus present interesting directions for future work on the role of effort outside the context of reward learning.

Finally, we have added a sentence to the Results section noting that participants very rarely failed to meet the required effort thresholds when registering their responses (page 13, lines 300-303):

We also confirmed that participants were able to successfully meet the required effort thresholds when registering responses in the experimental blocks (< 0.7% of trials missed per participant on average), and found that this did not differ significantly between drug groups (z = -1.46, p = .15).

Minor comments:

1. At a few points the authors call their task a 'reinforcement learning paradigm', which is not inaccurate but is very nonspecific (in principle, any task with reward and feedback can be modelled using RL). Rather, the task should probably be referred to as a two-armed bandit which is then modelled using reinforcement learning.

Thank you for pointing this out. We have now updated the revised manuscript to refer to the paradigm as a ‘two-armed bandit’ task throughout, and we use the term ‘reinforcement learning’ only when referring to the models.

2. I am unsure as to how Fig. 3C is related to motor performance. As the authors point out, the probability of choosing the low effort option is matched across the two groups, but isn't the more pertinent fact that they are always able to carry out the effort required as shown in 3B? (I am assuming the dotted lines in 3B relate to the threshold for successful effort exertion). 3C seems to speak more to whether participants are effort averse.

The Reviewer’s understanding is correct that these plots in Fig. 3 are intended to display different features of participant behaviour (motor capacity vs. effort aversion). We have now removed the Fig. 3 subheadings to avoid confusion. We have also updated the caption to explain that the dotted lines indicate minimum effort thresholds. Note that Fig. 3 has also been revised in response to another Reviewer’s suggestion to divide each plot into responses to the low vs. high effort stimulus. Revised Fig 3. (page 14):

***Fig 3. Motor and learning performance in the sulpiride and placebo groups.*** (A-C) Motor performance did not differ significantly between drug groups with respect to (A) MVCs, (B) mean effort exerted during the experimental blocks, or (C) standard deviation of effort during the experimental blocks. Dotted lines in (B) indicate minimum effort thresholds for the low and high effort stimulus, respectively. (D) Sulpiride did not affect preferences for low vs. high effort actions compared to placebo. (E-G) Learning performance differed between drug groups. Relative to placebo, participants on sulpiride exhibited (E) significantly lower choice accuracy (p = .044), and (F) were less likely to repeat rewarded choices (p = .005), but (G) were no different in their tendency to switch stimulus following non-rewarded choices (p = .4). In addition, across both drug groups, participants were less likely to repeat high effort choices than low effort choices, consistent with effort aversion (F-G). Statistical significance marks are shown for differences between drug groups. *p < .05; **p < .01; PL = placebo; SP = sulpiride.

Line 165-166: please be more explicit as to what a 'positively-valenced' (or negatively-valenced) auditory tone is. Is the pitch different, or something else?

These details have been added to the revised manuscript (page 7, lines 171-174):

On rewarded trials, a ‘smiley face’ was presented for 0.5 seconds accompanied by a positively-valenced auditory tone (‘cash register’ sound effect). On non-rewarded trials, a ‘sad face’ was presented for 0.5 seconds with a negatively-valenced tone (‘wrong answer buzzer’ sound effect).

We thank the Reviewer for their helpful comments and suggestions.

**Additional author correction**

In reanalysing the results on heart rate and blood pressure reported in the Supporting Information, we noted an error in the extraction of these data. Specifically, where we previously reported small differences in heart rate and blood pressure between drug groups, no significant group differences in fact exist. We note that this correction has no bearing on the main findings of the study. We have updated the manuscript accordingly (page 36, lines 943-954):

There was a significant main effect of Time on HR (F(4,160) = 29.05, p < .001), such that HR decreased over the first and second hours post-ingestion across both groups (0-1 and 1-2 hrs, both p ≤ .009; 2-3 and 3-4 hrs, both p > .99). HR did not differ significantly between Drug groups (main and interaction effects both ≥ 0.53).

We also observed a main effect of Time on sBP (F(3.3,131.93) = 4.87, p = .002), such that sBP decreased in the first hour post-ingestion (0-1 hrs, p = .003) and increased in the fourth hour post-ingestion (3-4 hrs, p = .044), with no other significant changes between time points (1-2 and 2-3 hrs, both p ≥ .74). There were no significant differences in sBP between Drug groups (main and interaction effects both p ≥ 0.28). Neither Time nor Drug significantly affected dBP (all p ≥ .24).

In sum, we found no effects of sulpiride on HR, sBP or dBP compared to placebo. Small variations in HR and sBP were detected over the course of the testing session, but these changes were seen across both drug groups and remained in the normal range.

Revised Fig. S2 (page 36):

***Fig S2. Drug effects on heart rate and blood pressure.*** Effects are plotted as a function of time post-ingestion of the sulpiride (red) or placebo (blue) capsules. bpm, beats per minute; sBP, systolic blood pressure; dBP, diastolic blood pressure.

**References**

Alexander, G. E. and M. D. Crutcher (1990). "Functional architecture of basal ganglia circuits: neural substrates of parallel processing." Trends Neurosci **13**(7): 266-271.

Bang, H., L. Ni and C. E. Davis (2004). "Assessment of blinding in clinical trials." Control Clin Trials **25**(2): 143-156.

Bennett, D., Y. Niv and A. J. Langdon (2021). "Value-free reinforcement learning: policy optimization as a minimal model of operant behavior." Curr Opin Behav Sci **41**: 114-121.

Bond, A. and M. Lader (1974). "The use of analogue scales in rating subjective feelings." British Journal of Medical Psychology **47**(3): 211-218.

Calabresi, P., B. Picconi, A. Tozzi and M. Di Filippo (2007). "Dopamine-mediated regulation of corticostriatal synaptic plasticity." Trends in neurosciences **30**(5): 211-219.

Calabresi, P., B. Picconi, A. Tozzi, V. Ghiglieri and M. Di Filippo (2014). "Direct and indirect pathways of basal ganglia: a critical reappraisal." Nature Neuroscience **17**(8): 1022-1030.

Carlsson, A. (1975). "Dopaminergic autoreceptors." Chemical tools in catecholamine research **2**: 219-225.

Chamberlain, S. R., U. Müller, A. D. Blackwell, L. Clark, T. W. Robbins and B. J. Sahakian (2006). "Neurochemical Modulation of Response Inhibition and Probabilistic Learning in Humans." Science **311**(5762): 861-863.

Collins, A. G. E. (2025). "A habit and working memory model as an alternative account of human reward-based learning." Nature Human Behaviour.

de Jong, J. W., S. A. Afjei, I. P. Dorocic, J. R. Peck, C. Liu, C. K. Kim, L. Tian, K. Deisseroth and S. Lammel (2019). "A neural circuit mechanism for encoding aversive stimuli in the mesolimbic dopamine system." Neuron **101**(1): 133-151. e137.

DeLong, M. R. (1990). "Primate models of movement disorders of basal ganglia origin." Trends Neurosci **13**(7): 281-285.

Eisenegger, C., M. Naef, A. Linssen, L. Clark, P. K. Gandamaneni, U. Müller and T. W. Robbins (2014). "Role of dopamine D2 receptors in human reinforcement learning." Neuropsychopharmacology **39**(10): 2366-2375.

Elsworth, J. D. and R. H. Roth (1996). "Dopamine autoreceptor pharmacology and function: Recent insights." The dopamine receptors: 223-265.

Emilien, G., J.-M. Maloteaux, M. Geurts, K. Hoogenberg and S. Cragg (1999). "Dopamine receptors—physiological understanding to therapeutic intervention potential." Pharmacology & Therapeutics **84**(2): 133-156.

Erfanian Abdoust, M., M. I. Froböse, A. Schnitzler, E. Schreivogel and G. Jocham (2024). "Dopamine and acetylcholine have distinct roles in delay- and effort-based decision-making in humans." PLoS Biol **22**(7): e3002714.

Ford, C. P. (2014). "The role of D2-autoreceptors in regulating dopamine neuron activity and transmission." Neuroscience **282**: 13-22.

Frank, M. J. and R. C. O'Reilly (2006). "A mechanistic account of striatal dopamine function in human cognition: psychopharmacological studies with cabergoline and haloperidol." Behav Neurosci **120**(3): 497-517.

Grace, A. A. (1991). "Phasic versus tonic dopamine release and the modulation of dopamine system responsivity: A hypothesis for the etiology of schizophrenia." Neuroscience **41**(1): 1-24.

Greenstreet, F., H. M. Vergara, Y. Johansson, S. Pati, L. Schwarz, S. C. Lenzi, J. P. Geerts, M. Wisdom, A. Gubanova, L. B. Rollik, J. Kaur, T. Moskovitz, J. Cohen, E. Thompson, T. W. Margrie, C. Clopath and M. Stephenson-Jones (2025). "Dopaminergic action prediction errors serve as a value-free teaching signal." Nature **643**(8074): 1333-1342.

Janssen, L. K., G. Sescousse, M. M. Hashemi, M. H. Timmer, N. P. ter Huurne, D. E. Geurts and R. Cools (2015). "Abnormal modulation of reward versus punishment learning by a dopamine D2-receptor antagonist in pathological gamblers." Psychopharmacology (Berl) **232**(18): 3345-3353.

Jarvis, H., I. Stevenson, A. Q. Huynh, E. Babbage, J. Coxon and T. T. Chong (2022). "Effort Reinforces Learning." J Neurosci **42**(40): 7648-7658.

Jocham, G., T. A. Klein and M. Ullsperger (2014). "Differential modulation of reinforcement learning by D2 dopamine and NMDA glutamate receptor antagonism." J Neurosci **34**(39): 13151-13162.

Linden, J., A. S. James, C. McDaniel and J. D. Jentsch (2018). "Dopamine D2 Receptors in Dopaminergic Neurons Modulate Performance in a Reversal Learning Task in Mice." eNeuro **5**(1).

Lopez, G. C. and T. N. Lerner (2025). "How dopamine enables learning from aversion." Current Opinion in Behavioral Sciences **61**: 101476.

Marcott, P. F., A. A. Mamaligas and C. P. Ford (2014). "Phasic dopamine release drives rapid activation of striatal D2-receptors." Neuron **84**(1): 164-176.

Matsuda, W., T. Furuta, K. C. Nakamura, H. Hioki, F. Fujiyama, R. Arai and T. Kaneko (2009). "Single nigrostriatal dopaminergic neurons form widely spread and highly dense axonal arborizations in the neostriatum." Journal of Neuroscience **29**(2): 444-453.

Miller, K. J., A. Shenhav and E. A. Ludvig (2019). "Habits without values." Psychol Rev **126**(2): 292-311.

Oleson, E. B., R. N. Gentry, V. C. Chioma and J. F. Cheer (2012). "Subsecond dopamine release in the nucleus accumbens predicts conditioned punishment and its successful avoidance." Journal of Neuroscience **32**(42): 14804-14808.

Shiner, T., B. Seymour, K. Wunderlich, C. Hill, K. P. Bhatia, P. Dayan and R. J. Dolan (2012). "Dopamine and performance in a reinforcement learning task: evidence from Parkinson's disease." Brain **135**(Pt 6): 1871-1883.

Soares-Cunha, C., B. Coimbra, A. David-Pereira, S. Borges, L. Pinto, P. Costa, N. Sousa and A. J. Rodrigues (2016). "Activation of D2 dopamine receptor-expressing neurons in the nucleus accumbens increases motivation." Nat Commun **7**: 11829.

Sommer, W. H., R. M. Costa and A. C. Hansson (2014). "Dopamine systems adaptation during acquisition and consolidation of a skill." Frontiers in Integrative Neuroscience **Volume 8 - 2014**.

Swainson, R., R. D. Rogers, B. J. Sahakian, B. A. Summers, C. E. Polkey and T. W. Robbins (2000). "Probabilistic learning and reversal deficits in patients with Parkinson's disease or frontal or temporal lobe lesions: possible adverse effects of dopaminergic medication." Neuropsychologia **38**(5): 596-612.

Takano, A., T. Suhara, F. Yasuno, K. Suzuki, H. Takahashi, T. Morimoto, Y. J. Lee, H. Kusuhara, Y. Sugiyama and Y. Okubo (2006). "The antipsychotic sultopride is overdosed--a PET study of drug-induced receptor occupancy in comparison with sulpiride." Int J Neuropsychopharmacol **9**(5): 539-545.

van der Schaaf, M. E., M. R. van Schouwenburg, D. E. M. Geurts, A. F. A. Schellekens, J. K. Buitelaar, R. J. Verkes and R. Cools (2014). "Establishing the Dopamine Dependency of Human Striatal Signals During Reward and Punishment Reversal Learning." Cerebral Cortex **24**(3): 633-642.

van Nuland, A. J., R. C. Helmich, M. F. Dirkx, H. Zach, I. Toni, R. Cools and H. E. M. den Ouden (2020). "Effects of dopamine on reinforcement learning in Parkinson’s disease depend on motor phenotype." Brain **143**(11): 3422-3434.
